# Current views on the role of Notch signaling and the pathogenesis of human leukemia

**DOI:** 10.1186/1471-2407-11-502

**Published:** 2011-11-30

**Authors:** Joanna Pancewicz, Christophe Nicot

**Affiliations:** 1 Department of Pathology, Center for Viral Oncology, University of Kansas Medical Center, Kansas City, KS 66160, USA

## Abstract

The Notch signaling pathway is highly conserved from *Drosophila *to humans and plays an important role in the regulation of cellular proliferation, differentiation and apoptosis.

Constitutive activation of Notch signaling has been shown to result in excessive cellular proliferation and a wide range of malignancies, including leukemia, glioblastoma and lung and breast cancers. Notch can also act as a tumor suppressor, and its inactivation has been associated with an increased risk of spontaneous squamous cell carcinoma. This minireview focuses on recent advances related to the mechanisms and roles of activated *Notch1*, *Notch2*, *Notch3 *and *Notch4 *signaling in human lymphocytic leukemia, myeloid leukemia and B cell lymphoma, as well as their significance, and recent advances in Notch-targeted therapies.

## Review

### Canonical and noncanonical activation of the Notch signaling pathway

The *Notch *gene was first described following the observation of Notches on the wings of fruit flies (*Drosophila melanogaster*) caused by partial loss of function of the *Notch *gene. Notch signaling is involved in many biological processes, ranging from embryonic development to cell proliferation and survival. It has been demonstrated that the Notch signaling pathway is involved in vascular formation and morphogenesis during vascular development. *Notch1*, *Notch2 *and *Notch4 *and its ligands (*Jagged1*, *Jagged2*, *Dll1 *and *Dll4*) are expressed in vascular endothelium, whereas *Notch3 *is expressed in vascular smooth muscle cells. Mutations in *Notch3 *are associated with CADASIL syndrome (cerebral autosomal dominant arteriopathy with subcortical infarcts and leukoencephalopathy), the human degenerative vascular disease.

**Figure 1:**
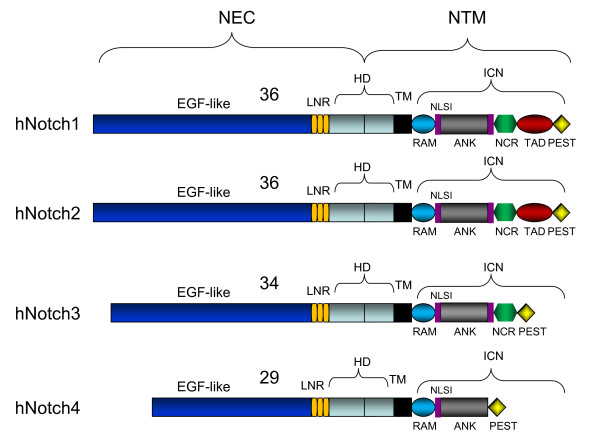
**Structure of the four human Notch receptors**. NEC: extracellular subunit; NTM: transmembrane subunit; EGF: epidermal growth factor; HD: heterodimerization domain; ICN: intracellular domain; LNR: cysteine-rich LNR repeats; TM: transmembrane domain; RAM: RAM domain; NLS: nuclear localizing signals; ANK: ankyrin repeat domain; NCR: cysteine response region; TAD: transactivation domain; PEST: region rich in proline (P), glutamine (E), serine (S) and threonine (T) residues.

**Figure 2:**
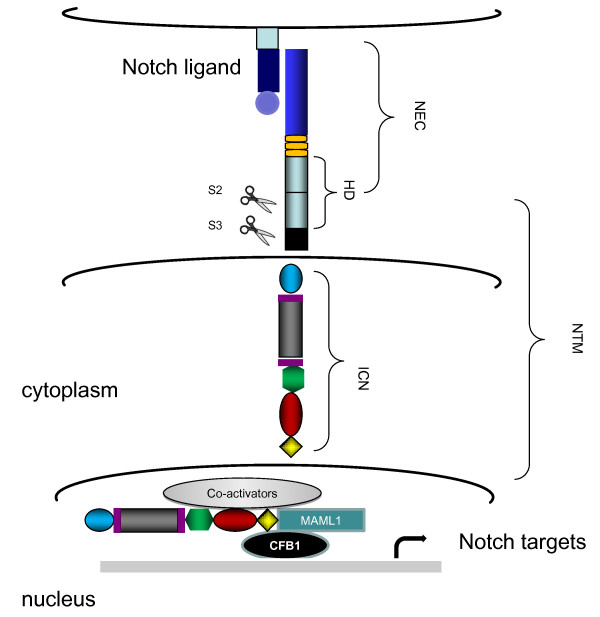
**The Notch signaling pathway**. The initiation of the Notch signaling pathway begins when the Notch ligand binds to the Notch receptor. This action triggers two proteolytic cleavages by ADAM-type protease (S2) and γ-secretase (S3), respectively. Following cleavages, the activated form of Notch is released (NICD) and is translocated to the nucleus, where NICD forms complexes with specific DNA-binding proteins (CBF1/Suppressor of Hairless/LAG-1 and Mastermind/SEL-8). Afterward the transcriptional process of target genes is initiated. MAML1: Mastermind-like 1 protein; CBF1: DNA-binding transcription factor.

**Figure 3:**
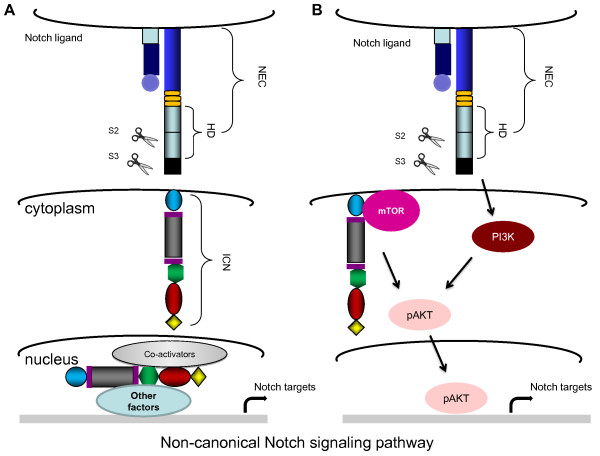
**Noncanonical Notch signaling pathway**. **(A) **NICD-dependent, CBF1-independent transcriptional activation by NICD, coactivators and other undefined factors **(B) **Interaction of NICD with components of other signaling pathways to activate Notch targets or tissue-specific factors.

The human Notch family includes four receptors and five ligands [1,2]. All four Notch receptors are synthesized as a single transmembrane polypeptide in the endoplasmic reticulum and transported to the cell surface trough the *trans*-Golgi network. Notch receptors are expressed as heterodimeric proteins with extracellular, transmembrane and intracellular domains (Figure 1). When a ligand of the Delta/Serrate/LAG-2 family (located on the surface of neighboring cells) binds to the extracellular domain of the Notch receptor, it triggers proteolytic cleavage by a metalloprotease (a disintegrin and metalloprotease (ADAM)). ADAM cleavage produces a substrate for a second cleavage by the presenilin-containing γ-secretase complex, releasing the Notch intracellular domain (NICD) [2,3] (Figure 2). NCID corresponds to the activated form of Notch, which translocates to the nucleus and forms complexes with specific DNA-binding proteins (CBF1/Suppressor of Hairless/LAG-1 and Mastermind/SEL-8) and transcriptionally activates target genes [4] (Figure 2). In the absence of receptor activation and NICD, CBF1 acts as a transcriptional repressor through interactions with the corepressors SMRT (silencing mediator of retinoid and thyroid receptors), KyoT2, CIR (CBF1-interacting corepressor) and SHARP (SMRT/HDAC1 (histone deacetylase 1)-associated repressor protein) [5]. In addition to canonical intracellular signaling pathways, there are other types of noncanonical Notch signaling (Figure 3). The first one involves Notch ligation and translocation of activation signals independent of CBF1 (NICD-dependent), the second involves activation of Notch target genes that are independent of γ-secretase cleavage (NICD- and CBF1-independent) and the third involves CBF1-dependent gene activation without receptor cleavage and NICD release. Termination of Notch signaling can occur at or downstream of the Notch receptor. The Notch receptor can be degraded through the lysosomes by the ubiquitin ligase Itch/AIP4 [6] or another ubiquitin ligase, Nedd4 [7], which act together with Numb [8] and Itch/AIP4 to stimulate endocytosis and lysosomal degradation of the Notch receptor [9]. Finally, NICD1 phosphorylation by GSK3 regulates its interaction with the E3 ubiquitin ligase CDC4/FBW7, thereby controlling NICD1 ubiquitination and proteasome-mediated degradation [10]. This multifaceted control of Notch expression underscores its critical functions in cellular homeostasis.

The role of the microenvironment in the activation of Notch in leukemia is increasingly recognized. Recently, *cis*-inhibition of Notch signaling by the DLL1 ligand has been described in *Drosophila *and mice [11]. These investigations have suggested that while expression of ligands on neighboring cells stimulates Notch activation, expression on the same cell as the Notch receptor may have an inhibitory effect [11]. Along these lines, activation of Notch signaling in B-cell malignancies might result from interactions between tumor cells as well as between the tumor cell and the microenvironment. There is evidence suggesting the importance of Notch signaling in the cross-talk between multiple myeloma (MM) cells and their environment. Bone marrow stromal cells express both Notch ligands, Jagged and δ, and are able to activate Notch signaling in MM cells [12,13].

### Mechanisms leading to constitutive activation of Notch signaling

*Notch1 *was discovered in humans through a t(7;9)(q34;q34.3) chromosomal translocation observed in some patients with T-cell acute lymphoblastic leukemia (T-ALL) [14,15]. However, a direct role of Notch activation in T-ALL remained obscure, since only 1% to 3% of patients with T-ALL were found to carry this translocation. It was only after the discovery of a high rate of activating mutations that it became clear that *Notch1 *expression is linked to the development of T-ALL [16]. Activating mutations identified in the T-ALL cluster at the heterodimerization domain (HD) and the proline, glutamine, serine and threonine (PEST) domain led to ligand-independent cleavage of the Notch receptor and a reduced degradation of NICD1, respectively. Recently, activating mutations in *Notch *were identified in more than 30% of human T-lymphotropic virus type I (HTLV-I)-associated adult T-cell leukemia (ATL) patients, suggesting an important role for Notch signaling in HTLV-I-associated ATL [17]. Activating mutations found in ATL patients are different from those previously reported in patients with T-ALL and mostly involve single-substitution mutations in the PEST domain that do not create an early stop codon, but rather lead to reduced CDC4/Fbw7-mediated degradation and stabilization of NICD1 [17].

Additional mechanisms have also been reported to lead to increased *Notch *expression in cancer cells. Mutations and internal duplication insertions in exon 28 of NICD [18], as well as mutations in *CDC4/Fbw7 *[10,19], have been reported, but they seem to occur at a very low frequency. The Wnt/β-catenin and Notch1 signaling pathways play an important role in a variety of biological processes, including cell proliferation and survival. Studies have shown that β-catenin can regulate the level and transcriptional activity of Notch1 [20]. β-catenin can prevent NICD degradation, possibly by competing with CDC4/Fbw7-dependent degradation [20]. Moreover, β-catenin increases the transcriptional activity of NICD, and the effects of β-catenin on Notch1 are noticeably reduced by overexpression of the lymphocyte enhancer-binding factor 1, LEF1.

### Implication of Notch signaling in various hematological disorders

#### Notch1

*Notch1 *has been reported to play a role in T-ALL and ATL, with approximate mutation rates of 50% and 30%, respectively [16,17,21,22]. *Notch1 *is required for the proliferation and survival of leukemia cells, and its role has been described in recent reviews [23-26]. The high prevalence of activating mutations found in *Notch1 *in T-ALL and ATL patients (Table 1) might suggest that this event plays a role in promoting the emergence of a particular subclone. Whether a mutation in Notch is a primary or secondary event in tumor cells is unclear. Mansour *et al*. [27] reported low-level *Notch1 *despite high blast counts in T-ALL patients, suggesting that these mutations were acquired as a secondary event in a preselected subclone. On the other hand, it has also been reported that mutation of *Notch1 *can be an early or initiating event in T-ALL arising prenatally, to be complemented by a postnatal *SIL*-*TAL1 *fusion gene and emergence of tumor clones [28].

**Table 1:** Mutation of *Notch1 *and *Notch2 *in human leukemia and/or lymphoma^a^

Leukemia and/or lymphoma type	Notch mutations in PEST/HD domain, %	Studies
*Notch1*		
T-ALL	50% PEST/HD	[16,49]
B-CLL	4.6% PEST	[50,51]
CLL	12.2% PEST	[52]
AML	8.3% PEST	[53]
T-NHL	42.9% PEST/HD	[49]
ATL	30% PEST	[17]
*Notch2*		
DLBCL	8% PEST	[31]
MZL	5% PEST	[32]

^a^PEST: proline, glutamine, serine, and threonine; HD: heterodimerization domain; T-ALL: T-cell acute lymphoblastic leukemia; B-CLL: B-cell chronic lymphocytic leukemia; CLL: chronic lymphocytic leukemia; AML: acute myeloid leukemia; T-NHL: T-cell non-Hodgkin lymphoma; ATL: adult T-cell leukemia; DLBCL: diffuse large B-cell lymphoma; MZL: marginal zone lymphoma.

#### Notch2

Increasing evidence suggests that *Notch2 *may play a role in leukemia and lymphoma. Early studies showed that feline leukemia virus recombinant genomes isolated from lymphomas captured *Notch2*, which included the intracellular ankyrin repeat functional domain in the envelope gene [29]. Later it was found that *Notch2 *plays a role in CD8 thymocyte maturation and that enforced expression of activated *Notch2 *invariably resulted in T-cell leukemia in mice [30]. Table 1 shows that approximately 8% of diffuse large B-cell lymphomas (DLBCLs) have *Notch2 *mutations [31]. Similar to observations in *Notch1*, mutations in DLCBL affected the PEST domain or a single-amino acid substitution at the C terminus and resulted in *Notch2*-reduced turnover [31]. These observations suggest *Notch2 *gain-of-function mutations in a subset of B-cell lymphomas. In fact, *Notch2 *is involved in the development of B1 and marginal zone B cells, and *Notch2 *is overexpressed in some marginal zone lymphomas (MZLs) [32]. Potential activating mutations of human *Notch2 *presented in Table 1 were also detected in 5% of MZL patients [32]. *Notch2 *may also play an indirect role in chronic B-cell lymphocytic leukemia (B-CLL) through upregulated expression of CD23 [33,34].

#### Notch3

A possible role of *Notch3 *in leukemia was postulated in studies in which transgenic mice expressing the constitutively active intracellular domain of *Notch3 *in thymocytes and T cells developed early and aggressive T-cell neoplasia [35]. Importantly, these results were validated in humans, and examination of T-ALL patients demonstrated high expression of *Notch3 *and *pTα *transcripts, whereas the expression of these genes was considerably reduced in or absent from patients in remission [36]. *pTα *and *Notch3 *interactions are essential for distribution of the E3 ligase protein, c-Cbl, to the lipid rafts. This is important in the development of leukemogenesis, since in the absence of *pTα*, c-Cbl targets *Notch3 *for proteasome degradation [37]. Moreover, the NF-κΒ pathway may be involved in the development of *Notch3*-dependent T-cell lymphoma in humans, and there is genetic and biochemical evidence that *Notch3 *triggers multiple NF-κΒ activation pathways [38]. Recently, *Notch3 *was found to control expression of mitogen-activated protein kinase phosphatase 1 and plays a role in the survival of T-ALL cells [39].

#### Notch4

*Notch4 *is expressed in human bone marrow cells and in CD34+ and CD34- populations [40]. *Notch4 *intracellular domain-transduced cord cells transplanted into mice showed remarkably elevated levels of engraftment of an immature T-cell population, while B-cell development was inhibited. Taken together, these results suggest that activation of *Notch4 *leads to enhanced stem cell activity, reduced differentiation and altered lymphoid development [41].

### Clinical relevance and therapeutic approaches aimed at targeting Notch signaling

The molecular pathogenesis of Notch has recently been reviewed [24]. Targeting Notch receptor cleavage through γ-secretase inhibitors (GSIs) is an attractive approach, as GSI treatment inhibits proliferation of T-ALL and ATL tumor cells *in vitro *and *in vivo *[42,43]. The use of GSIs, however, poses several challenges. Current GSIs have been shown to have significant intestinal toxicity in patients because of the dual inhibition of *Notch1 *and *Notch2*. In addition, high levels of IL-6 and IL-8 have been reported to abrogate or significantly reduce the efficacy of the GSI (RO4929097) [44]. GSIs may not be useful in patients with a *Notch1 *mutation in the HD, as this results in weakened association or complete dissociation of the receptor subunits and thus leads to ligand-independent activation. Finally, recent studies have also shown that T-ALL patients with loss of *PTEN *are resistant to GSI effects [45]. Together these results highlight the need for new targeted therapies.

Recently, the use of therapeutic antibodies that selectively block Notch1 receptor signaling has been reported to inhibit tumor growth in mouse models [46]. Rather than targeting receptor processing, alternative strategies may also focus on blocking NICD functions and transcriptional activities. Along these lines, the use of a stapled peptide to inhibit the Notch transcription factor complex has been reported to result in Notch-specific antiproliferative effects in cultured cells and in a mouse model of Notch1-driven T-ALL [47].

## Conclusions

Increased Notch signaling is linked to hematological malignancies. Overexpression of activated the *Notch1*, *Notch2 *or *Notch3 *genes in bone marrow progenitor cells reliably induces T-ALL at high frequency in murine models. Activating mutations in *Notch1 *is frequent in both T-ALL and ATL. It has been proposed that a fraction of T-ALLs that present as leukemia without evidence of thymic involvement may originate from bone marrow progenitors that have acquired a *Notch1 *mutation, suggesting that constitutive active *Notch1 *could, in some cases, be an initial event driving tumor development. On the other hand, animal model studies have indicated that *Notch *can also play a secondary role during T-ALL development. The role played by *Notch1 *in other human leukemias is less clear, as is the potential role of other *Notch *genes. Although inhibition of Notch signaling in xenograft tumors in animal models effectively prevents tumor cell growth, clinical outcomes in humans do not seem to be significantly associated with *Notch *status, which has an impact on early response to treatment but not on general outcomes in pediatric patients with T-ALL [48]. This may be related to acquisition of additional mutations in patients who have received several therapies and may relieve tumor cells from Notch1 addiction. As is the case with many targeted therapies, resistance to single drugs emerges rapidly, suggesting that a multidrug chemotherapy targeting Notch and connected pathways is needed.

## Abbreviations

IL: interleukin; NF-κΒ: nuclear factor κΒ.

## Competing interests

CN is a section editor for *BMC Cancer*.

## Authors' contributions

JP created the figures and wrote the manuscript. CN wrote the manuscript. Both authors read and approved the final manuscript.

## Pre-publication history

The pre-publication history for this paper can be accessed here:

http://www.biomedcentral.com/1471-2407/11/502/prepub
